# Impact of COVID-19 on Dental Care during a National Lockdown: A Retrospective Observational Study

**DOI:** 10.3390/ijerph18157963

**Published:** 2021-07-28

**Authors:** Elias Walter, Leonard von Bronk, Reinhard Hickel, Karin Christine Huth

**Affiliations:** Department of Conservative Dentistry and Periodontology, University Hospital, LMU, 80336 Munich, Germany; lvonbron@dent.med.uni-muenchen.de (L.v.B.); Hickel@dent.med.uni-muenchen.de (R.H.); khuth@dent.med.uni-muenchen.de (K.C.H.)

**Keywords:** COVID-19, urgent dental care, lockdown, pandemic

## Abstract

The coronavirus disease 19 (COVID-19) has challenged dental health professions. This study analyzes its impact on urgent dental care in the Department of Conservative Dentistry and Periodontology, University Hospital Munich and Bavaria, Germany. Patient numbers without and with positive/suspected COVID-19 infection, their reasons for attendance, and treatments were retrospectively recorded (February–July 2020) and linked to local COVID-19 infection numbers, control measures, and numbers/reasons for closures of private dental practices in Bavaria, Germany. Patient numbers decreased within the urgent care unit and the private dental practices followed by a complete recovery by the end of July. While non-emergency visits dropped to almost zero during the first lockdown, pain-related treatments were administered invariably also in patients with positive/suspected COVID-19 infections. Reasons for practice closures were lack of personal protective equipment (PPE), lack of employees, staff’s increased health risks, and infected staff, which accounted for 0.72% (3.6% closures in total). Pain-driven urgent dental care remains a constant necessity even in times of high infection risk, and measures established at the beginning of the pandemic seem to have provided a safe environment for patients as well as oral health care providers. PPE storage is important to ensure patients’ treatment under high-risk conditions, and its storage and provision by regulatory units might guarantee a stable and safe oral health care system in the future.

## 1. Introduction

Emerging infectious diseases (EID) have increased in the last century due to socio-economic factors such as population density and global traffic [1]. One of those pathogens, a novel coronavirus causing the severe acute respiratory syndrome (SARS-CoV-2) [2], emerged at the end of the year 2019 in Wuhan, China. The resulting coronavirus disease 19 (COVID-19) developed quickly toward a global pandemic and health crisis. After a year, it is still ongoing, with over 300,000 reported cases per day worldwide and over 2,500,000 deaths in total as of 1 March 2021 [3]. The first patient in Germany was diagnosed in Munich, and the city quickly became a hotspot in the country [4]. Similar to many other nations, the number of infections was reduced by, so far, two national lockdowns, which were accompanied by recommendations to suspend elective medical/dental care [5,6]. Such extensive measures are unprecedented in modern history and have unpredictable effects on medical specialties and their patients. Additionally, transmission via aerosols and droplets [7,8], which are unavoidable for many dental procedures, as well as proximity to and number of patients attended by dental staff comprise a high risk of infection.

Nevertheless, dentistry has been considered an essential service during the pandemic, but its impact on the profession and patients has not been evaluated yet [6]. The first infection wave was followed by so far two others with higher infection numbers. Even though vaccines became available in January 2021, the population remains in a state of uncertainty because of several new COVID-19 variants [9,10]. This uncertainty in the development of the pandemic calls for a detailed analysis of demand for as well as specifics of dental treatment during the first infection wave in order to predict and plan dental care in the future [11]. Accordingly, a recent article asked for a consensus on the definition of essential oral health care and postulated a model with advanced, essential, basic, and urgent care [12].

The urgent care unit of the Department of Conservative Dentistry and Periodontology is one of the primary diagnosis centers for acute dental problems at the University Hospital Munich. At the beginning of the pandemic, a detailed report about the department’s safety measures against COVID-19 was published, but the effects and efficiency have not been evaluated yet. In the presented study, we analyzed patient development during the pandemic and evaluated the necessity of dental treatments. Therefore, the aim of this retrospective study was to record patient numbers without and with positive/suspected COVID-19 infections, their reasons for attendance, and associated treatments, and to link these data to local COVID-19 infection numbers, control measures, as well as numbers and reasons for the closures of private dental practices in Bavaria, Germany.

## 2. Materials and Methods

### 2.1. Study Design and Setting

This retrospective observational surveillance study focused on the urgent outpatient care unit of the aforementioned department between February 1 and the end of July 2020. February was considered as baseline, as the public awareness toward COVID-19 was low. Patients could attend the urgent care unit spontaneously without appointment or prerequisites. Primary care was carried out. If necessary, patients were sorted to specific units for further care later on. The urgent care unit provides an optimal and stable setting to analyze patients’ flow before, during, and after the national lockdown. Other subunits within the same department—namely, the private outpatient care, pediatric dentistry, or special care—were not included in this study because their treatment spectrum is more complex. The teaching section switched from patient treatment to phantom head simulation to guarantee students safety and therefore was omitted. Additionally, the outpatient care of the oral and maxillofacial surgery and prosthodontics departments were not considered in this study. In comparison, the mean patient numbers during the same calendar weeks in the years from 2016 to 2019 were collected. The study was approved by the medical faculty ethics committee of the Ludwig-Maximilians-Universität, Munich (No. 20-0904).

A detailed description of measures regarding the increased hygienic demands and requirements for social distancing during the COVID-19 pandemic has been given before [13]. In short, each patient was assessed for the likeliness of SARS-CoV-2 infection by a questionnaire before entering the clinic. They were asked if they presented symptoms that coincide with COVID-19, if they had traveled to a COVID-19 risk area, or if they have had close contact to a positive COVID-19 patient. Additionally, temperature was measured at a triage station at the entrance of the clinic. Upon a positive answer or increased temperature, the respective patient was treated in a separated unit by staff wearing increased personal protective equipment (PPE) [13]. During that time period, the testing infrastructure was not as advanced, and COVID-19 confirmation could often not be obtained before treatment in the urgent care unit. All other patients were treated under regular dental protective measures.

### 2.2. Data Acquisition

All patients were asked for their reason of attendance before being admitted to the urgent care section. These reasons were recorded and afterwards categorized in pain, broken tooth or restauration, trauma, continued appointment, and checkup regarding simple questions such as dentin hypersensitivity. To date, these reasons represented the opinion/perception of each patient on their need of dental care regardless of professional dental diagnosis or the following treatment.

The following treatments were categorized into endodontic, restorative, periodontal, surgical, trauma, or pharmacological intervention. Each patient received a checkup, and patients without the need of immediate intervention were categorized into checkup. Multiple answers were possible if separate treatments independently of each other were needed in one patient.

A distinct group of patients was referred from other medical departments, for example for identification of unknown infection origins, before radio- or chemotherapy or general dental problems during the patients’ hospital stay. These were summarized by the category “inpatient consultation” regarding both reason of attendance as well as treatment. All data were collected in October 2020.

To complete the picture of the necessity of urgent dental care, the following additional data were collected: first, the number of positively tested COVID-19 cases in Munich according to the Robert Koch Institute (RKI) and the national health department in Germany [14] (collected 24 October 2020); second, governmental measures taken in Bavaria for infection control [15] (collected 24 October 2020); third, information about the number and reasons of practice closures in Bavaria and the amount of quarterly invoiced treatments in comparison to 2019 were kindly provided by the Bavarian Association of Statuary Health Insurance (KZVB) (15 December 2020). This was possible as practices that treat patients insured by the statutory health insurance are obliged to report closures to the KZVB. Reasons for closure were categorized into missing PPE, missing staff, administrative order, and others. Multiple answers were possible.

### 2.3. Statistics

The software Microsoft Excel (16.43) (Microsoft, Redmond, WA, USA) was used for descriptive data analysis. Data were depicted in bar diagrams and pie charts using Photoshop 2020 (21.2.2) and Illustrator 2020 (24.3) (both Adobe, San Jose, CA, USA). For statistical analysis, data were grouped into pre-lockdown (3 February 2020 to 21 March 2020), lockdown (22 March 2020 to 25 May 2020) as well as post-lockdown (26 May 2020 to 31 July 2020). Mean and standard deviation were calculated for each group and statistically compared (Prism, GraphPad Software, San Diego, CA, USA). Gaussian distribution was tested by Shapiro–Wilk tests. If data were distributed normally, one-way ANOVA was used in conjunction with Bonferroni correction. Otherwise, the Kruskal–Wallis test with Dunn’s correction was used to determine significance. Significance was assumed at *p* ≤ 0.05.

## 3. Results

### 3.1. Patient Numbers during the COVID-19 Pandemic

**Figure 1:**
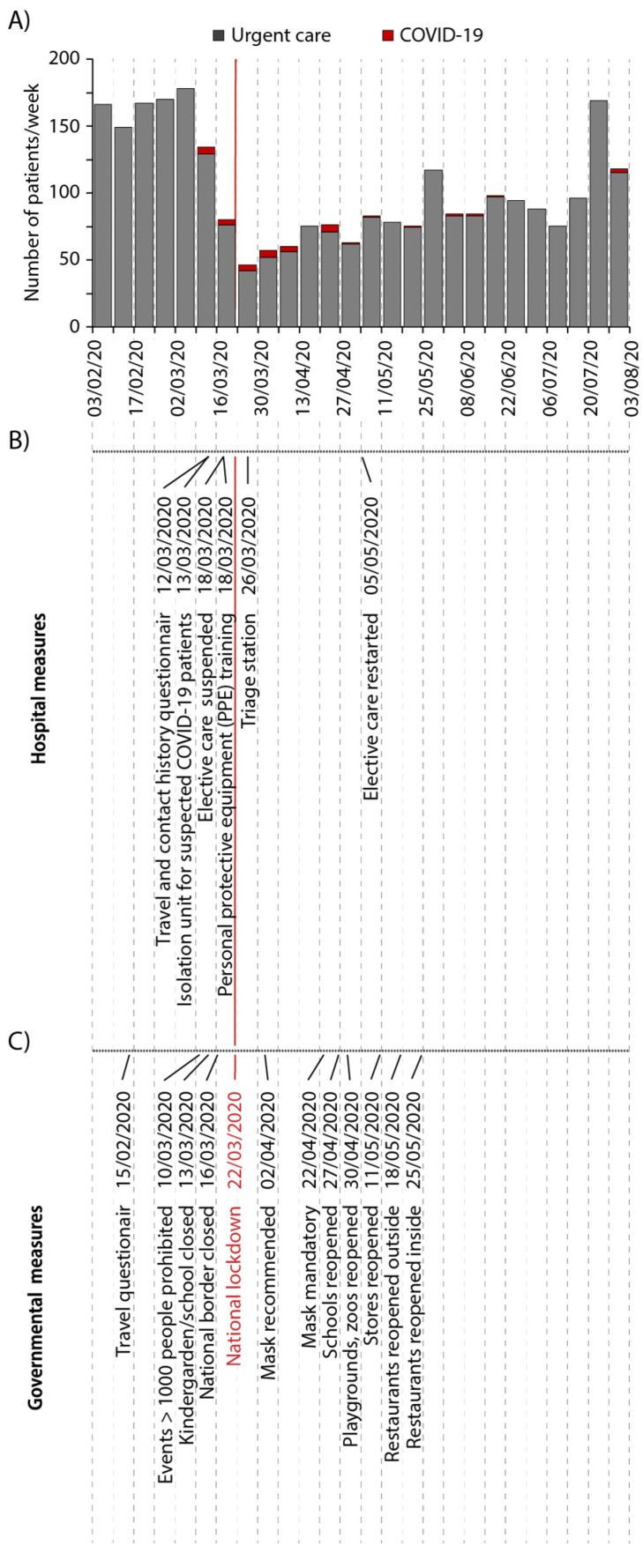
Patient numbers in the urgent dental care unit along with measures for infection control during the initial period of the COVID-19 pandemic. (**A**) Weekly patient numbers in the urgent dental care unit and patients with positive/suspected COVID-19 infections from 3 February to 31 July 2020. In temporal context, the middle and lower section show corresponding (**B**) hospital and (**C**) governmental measures taken as precautions against COVID-19. Each measure is given on the timeline (dotted line, each dot represents a day with weekly separation by vertical lines). The red line marks the beginning of the national lockdown.

**Figure 2:**
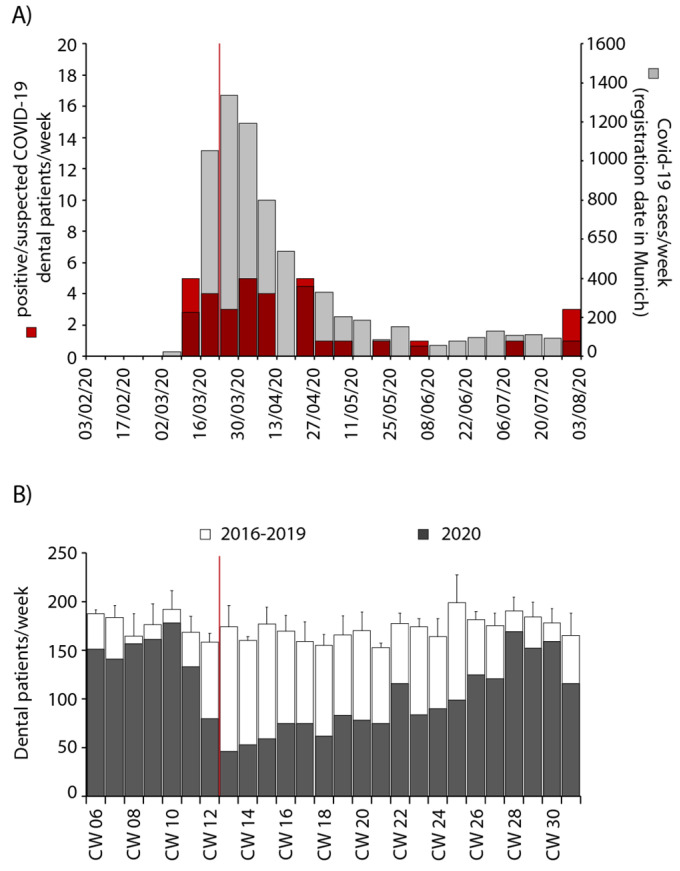
Number of patients with positive/suspected COVID-19 infections concurrent with COVID-19 infections in Munich and comparison of patient numbers with previous years. (**A**) Patients with positive/suspected COVID-19 infections solely in the urgent dental care unit per week (left axis, red) in comparison with officially registered COVID-19 cases in Munich per week (right axis, grey) from 3 February to 31 July 2020. (**B**) Total patient numbers per week compared with patient numbers of the years 2016–2019 in the same calendar weeks (CW, mean ± standard deviation). The red line marks the beginning of the first national lockdown.

Firstly, the effect of the COVID-19 pandemic on patient volume was recorded (Figure 1A) along with measures for infection control by the University Hospital (Figure 1B) as well as the German government (Figure 1C). In total, 3014 patients attended the urgent care unit between 3 February 2020 and 31 July 2020. Of these, 51% were male and 49% were female with an age range from 2 to 94 years and a median age of 54 years (Figure S1). The public awareness of SARS-CoV-2 at the beginning of March led to a quick decline in patient numbers. On 18 March, elective treatments were suspended by governmental order, while urgent dental care was maintained [13]. At this time, COVID-19 cases increased exponentially in Munich, and accordingly, the number of positive/suspected COVID-19 patients raised to a maximum of 5 patients with an average of 3.74 ± 1.67 per week for 6 weeks (Figure 2A). The following week after the national lockdown showed the lowest number of patients receiving urgent dental care with only 46 patients (23 March to 30 March). This constitutes only 25% of the average patient volume in previous years (Figure 2B) and coincided with the government ordering the national lockdown on 22 March. The peak of COVID-19 cases was reached 8 days after introduction of the lockdown with 314 reported new cases per day in Munich on 31 March.

Within the following three months, the number of patients recovered slowly. Accordingly, reported COVID-19 cases per day in Munich declined. During this time, elective treatments restarted on 5 May, 48 days after switching to solely urgent care, while positive/suspected COVID-19 patients declined to one every couple of weeks (Figure 2A). At this time, on average, 15 cases of COVID-19 were reported per day in Munich, and lockdown measures were loosened by the government and restaurants and stores were reopened. At the end of July, patients receiving urgent dental care normalized to around 30 a day, similar to previous years, which showed a stable average of approximately 35 patients (Figure 2B).

### 3.2. Reasons for Seeking Urgent Dental Care and Resulting Treatment

**Figure 3:**
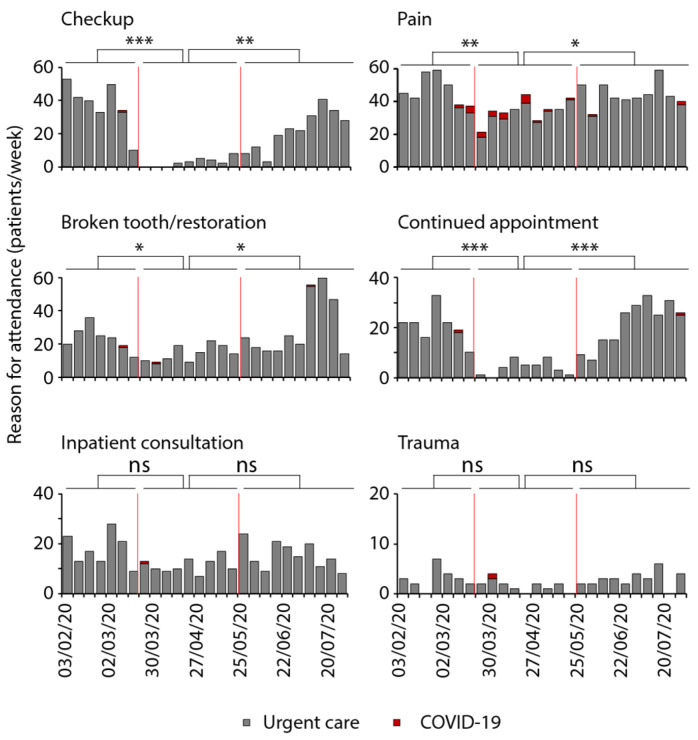
Reasons for seeking urgent dental care during the initial period of the COVID-19 pandemic. Patients´ reasons for attending the urgent dental care unit were categorized into checkup with minor questions (*n* = 507), pain (*n* = 1079), broken tooth/restauration (*n* = 588), continued appointment (*n* = 395), inpatient consultation (*n* = 381), and trauma (*n* = 64) from 3 February to 31 July 2020. The first red line marks the beginning and the second marks the end of the national lockdown, dividing the time into pre-lockdown, lockdown, and post-lockdown. The red bars represent patients with high-risk of or confirmed COVID-19 infection (ANOVA; * *p* ≤ 0.05, ** *p* ≤ 0.01, *** *p* ≤ 0.001).

The reasons given by the patients for attending the urgent dental care unit were recorded weekly (Figure 3). In February 2020, which was considered as baseline, these reasons were pain (25%), checkups for minor questions (26%), and broken restorations or broken tooth (12%). Inpatient consultations accounted for 17% of all reasons; 15% of the patients came for continued treatment, which had been started earlier. Acute dental trauma constituted 5% of the reasons.

From March onwards, when the broad public became aware of the pandemic, the patient numbers decreased, and elective treatments including checkups with minor questions or second opinions were suspended, resulting in distribution changes of the remaining reasons. Continued appointments and checkups decreased to zero directly after the lockdown and only recovered slowly. Only pain as a reason remained constant with approximately 30 patients per week, which accounted for more than 50% of all patients during the lockdown. Broken teeth or insufficient restoration as a reason decreased slightly but recovered fast with a slight overcompensation at the beginning of July for three weeks. Inpatient consultations were only mildly affected.

**Figure 4:**
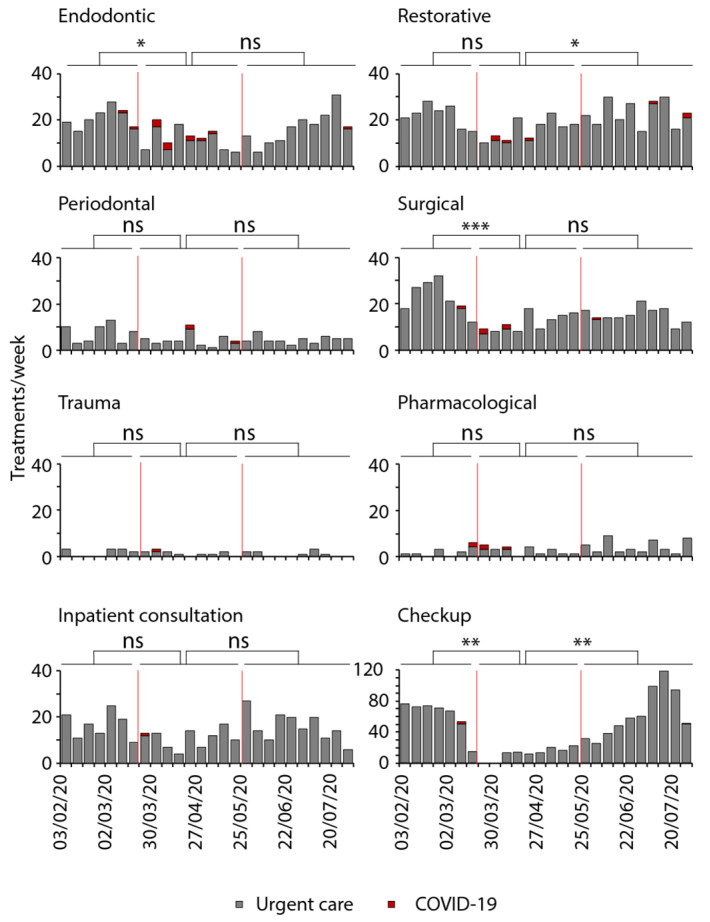
Dental treatments carried out in the urgent dental care unit during the initial period of the COVID-19 pandemic. Treatments performed on patients per week in the urgent dental care unit from 3 February to 31 July 2020. Treatments were categorized into endodontic (*n* = 410), restorative (*n* = 525), periodontal (*n* = 137), surgical (*n* = 410), trauma (*n* = 32), and pharmacological (*n* = 77) interventions as well as inpatient consultations (*n* = 370) or checkups with minor questions (*n* = 1160). The first red line marks the beginning and the second marks the end of the national lockdown, dividing the time into pre-lockdown, lockdown, and post-lockdown. The red bars represent patients with high risk of or confirmed COVID-19 infection (ANOVA or Kruskal–Wallis; * *p* ≤ 0.05, ** *p* ≤ 0.01, *** *p* ≤ 0.001).

In the following, the actual treatments were recorded and categorized (Figure 4). In February (baseline), checkups without urgent interventions were the most prevalent (45%). Restorative (15%), endodontic (13%), as well as surgical (17%) treatments were applied to a similar extent. Less common were acute periodontal therapy (5%) and acute trauma treatment (1%). Inpatient consultations were applied in 14% of all patients.

Directly after the lockdown, checkups without practical intervention dropped abruptly from 45% to zero (Figure 4), meaning that all patients who attended the urgent care unit until 21 April needed urgent interventions. In detail, endodontic therapy was necessary on average in 19 patients per week before the lockdown, and it decreased afterwards by 42% to 11 patients on average per week by 15 June and returned to initial numbers thereafter. Nevertheless, endodontic therapy was the least affected treatment. Restorative therapy decreased from 21 patients per week before the lockdown by 55% to 10 patients afterwards and increased again to normal levels by 25 May with 22 patients per week on average. Similarly, surgical treatment decreased from 24 patients on average to eight patients after the lockdown (67%) but did not recover fully in the recorded timeframe. In July, only an average of 14 patients required surgical treatment per week. In contrast, acute periodontal therapy as well as trauma treatments remained constant. Inpatient consultations also slightly decreased after the lockdown, assumingly because the referring medical specialties were affected by COVID-19 as well and therefore required less dental expertise. After 25 May, these numbers recovered to similar numbers as before (average 15 patients weekly).

### 3.3. Treatments Performed on Patients with Positive/Suspected COVID-19 Infections

**Figure 5:**
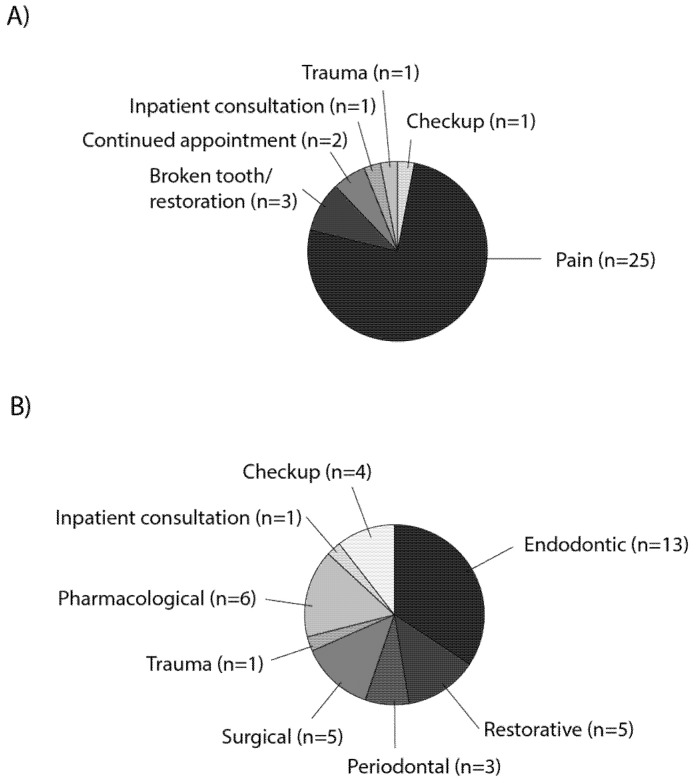
Reasons of attendance given by patients with positive/suspected COVID-19 infections and respective dental treatments. Pie charts depicting (**A**) reasons of attendance and (**B**) treatments of patients with positive/suspected COVID-19 infections from 3 February to 31 July 2020.

Solely within the urgent dental care unit, 33 patients with positive/suspected COVID-19 infections were registered between February and July, of which the majority (25 patients) attended during the period of highest COVID-19 prevalence in Munich in between 9 March and 27 April. Twenty-five of the 33 patients showed severe dental pain, while all other reasons were sparsely represented (broken tooth/restoration: 3, continued appointment: 2, inpatient consultation: 1, trauma: 1, checkup: 1) (Figure 5A). The resulting treatments were endodontic in most cases (13), whereas all other categories appeared less than six times each (Figure 5B). In only four COVID-19 patients urgent intervention was not necessary, which was in early March, when emergency protocols were not yet fully established.

### 3.4. Effects of COVID-19 Reported for Dental Care in Bavaria

**Figure 6:**
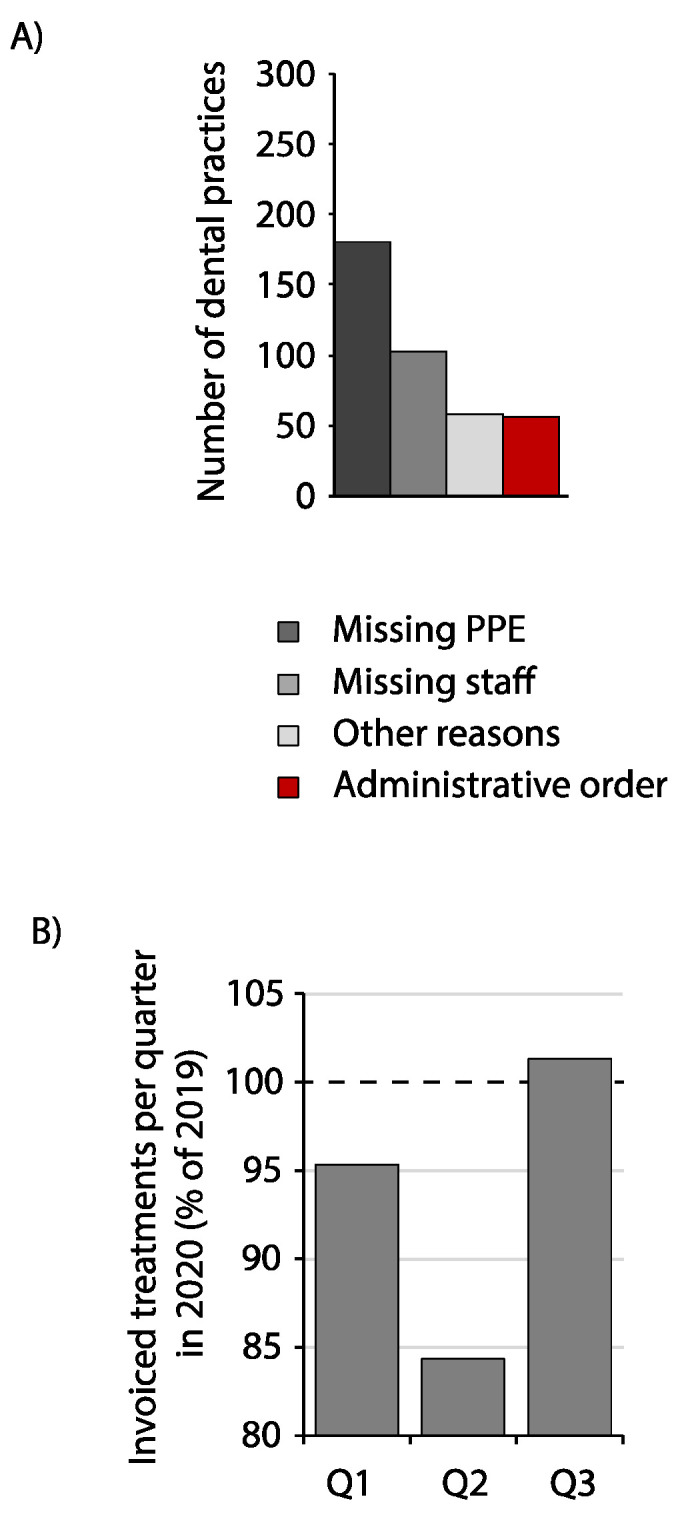
Effects of the COVID-19 pandemic on Bavarian dental practices. (**A**) Reason for temporary closure of dental practices in absolute numbers on 6 April 2020. (**B**) Invoiced treatments of patients insured under a statutory insurance in Bavaria in the quarters Q1–Q3 in 2020 in percent of the same quarters in 2019.

The Bavarian Association of Statuary Health Insurance (KZVB) reported that during the first national lockdown, up to 3.6% of their contractual dental practices were closed. Out of these, 0.72% were closed by governmental order because of a positive COVID-19 diagnosed staff member. On 11 May, only 0.16% closures were still recorded in Bavaria. As reasons for closure, 66% of all closed dental practices stated missing proper PPE including regular facemasks and gloves, 38% reported lack of employees, and 20% reported health risk factors such as immunosuppression or hearth disease during the whole timeframe (Figure 6A).

The quantity of invoiced treatments in Bavarian practices regarding patients with public insurance may be indicative of the impact of the pandemic on dental care in Bavaria. Accordingly, a decrease of those invoiced treatments of 4.7% was reported by the KZVB for the first quarter of 2020 (January to March) compared to the same quarter in 2019 (Figure 6B). In the second quarter (April to June), a reduction of 15.4% was registered, which comprised a reduction of patient numbers as well as treatments during the first lockdown. The third quarter (July to September) showed a recovery with 101.3% of the same quarter in 2019.

## 4. Discussion

During the first six months of the pandemic, a drop in patients was observed in the urgent dental care unit of the Department of Conservative Dentistry and Periodontology in Munich as well as in Bavarian dental practices as reported by the KZVB. A similar decrease in patients was observed in several countries [16,17]. In March, this drop was particularly evident for routine checkups and elective treatments that were not pain driven. Fear of contracting COVID-19 at the dentist as well as recommendations to suspend elective dental care by regulatory institutions during the first lockdown have been reported as reasons [6,18,19]. However, routine checkups are essential for preventive dentistry and are associated with increased oral health [20,21], whereas pain-triggered visits are associated with poorer oral-health-related quality of life [22]. For example, in Switzerland, routine dental checkups constitute 33% of all dental appointments under normal circumstances [23], corresponding to 26% routine checkups including minor questions of all visitations in the present study before the public awareness of COVID-19 in February 2020 (baseline). In the United States, a similar decrease in routine visits was observed; however, it was accompanied by an increase in tooth extractions, which has been attributed to loss of insurance due to increasing unemployment [24].

As a consequence, it may be assumed that the pandemic could have long-term effects on the prevention and progression of oral diseases. This is underlined by the fact that only patients with acute pain remained constant, of which almost all received urgent interventions. Less acute but nevertheless existing dental problems were neglected [25,26], which was mainly noticed by a reduction in restorative, endodontic, as well as surgical interventions during the first lockdown. This change could have impaired people’s oral health within the respective time period and emphasizes the importance of a working oral health care system during a pandemic. It even has been reported that urgent interventions could not be administered because of limited access to dental practices [27]. In the beginning of the lockdown in Bavaria, the lack of sufficient basic PPE was the most common reason for closures of dental practices. Therefore, the provision and storage of PPE could help to avoid such closings in order to guarantee dental care during acute infection waves. Additionally, during the first lockdown, testing infrastructure was limited, and upon suspected COVID-19 infection, patients were required to be treated as such. This access limitation is especially true for rural areas, private dental practices, or upon the emergence of new diseases.

Therefore, it has been suggested to implement a travel-, contact- and symptom-history questionnaire to screen for positive COVID-19 cases from the beginning of the pandemic [13,16,28]. Interestingly, the peak of registered patients with positive/suspected COVID-19 infections in the urgent dental care unit of our department coincided with the peak of infection numbers in Munich during the first wave, indicating the effectiveness and importance of such screening methods. In addition, telephone screening has been evaluated to reduce the number of dispensable dental visits effectively [29].

A systematic review has described gustatory dysfunctions and rarely oral mucosal lesions in COVID-19 positive patients [30]. In the urgent care unit, these symptoms were not the reason for seeking dental care but underlying preexisting dental problems that might have been avoidable by preventive checkups.

For the treatment of these patients with positive/suspected COVID-19 infections, more intensive protection protocols for staff members have been indicated [4]. Although the risk of transmission has been evaluated to be very high for dental staff due to aerosol generating dental procedures and proximity to the respiratory tract during treatments [8,31,32], dental offices have not revealed higher infection numbers. Accordingly, an analysis of oral health care workers in Wuhan showed only few COVID-19 infections in dental staff, which could not be traced back to dental treatment itself [16]. During the first infection wave in April, only 0.72% of dental practices in Bavaria reported a positive COVID-19 infection of a staff member. A similar low percentage has been reported for dentists in the United States [33]. Accordingly, no staff contracting COVID-19 during work has been registered in our department so far nor have SARS-CoV-2-antibodies been found in July 2020 (unpublished study). Only three staff members contracted COVID-19 by 19 July 2021, and all could be traced back to private contacts. These data show that protocols established at the beginning of the pandemic [13] in conjunction with already high hygiene standards seem to provide a safe environment for oral health practitioners as well as patients.

## 5. Conclusions

In conclusion, accessibility of dental care is necessary even under circumstances of high infection risk, and measures should be taken to guarantee its continuation. Data showed a distinct number of patients with positive/suspected COVID-19 infection needed predominantly pain-related urgent dental interventions. Measures established at the beginning of the pandemic include careful detection of patients with high COVID-19 risk and treatment of those with advanced PPE, which seem to have provided a safe environment for patients as well as oral health care providers. The storage as well as provision of PPE to dental practices by regulatory units could help to guarantee a stable and safe oral health care system in the future.

## Data Availability

The data that support the findings of this study are available on reasonable request from the corresponding author.

## Supplementary Materials

The following are available online at https://www.mdpi.com/article/10.3390/ijerph18157963/s1, Figure S1: Dotplot of patients‘ age in urgent care pre-lockdown, during lockdown as well as post-lockdown.

## Abbreviations

PPE	Personal protective equipment
EID	Emerging infectious disease
SARS-CoV-2	Severe acute respiratory syndrome coronavirus 2
COVID-19	Coronavirus disease 19
