# Supplementary material for: Impact of COVID-19 on Dental Care during a National Lockdown: A Retrospective Observational Study
Source: Int J Environ Res Public Health. 2021 Jul 28;18(15):7963. doi: 10.3390/ijerph18157963 (PMC8345748; doi:10.3390/ijerph18157963)
Supplement: Supplementary file 1 [file ijerph-18-07963-s001.zip › ijerph-1298779-supplementary.pdf]

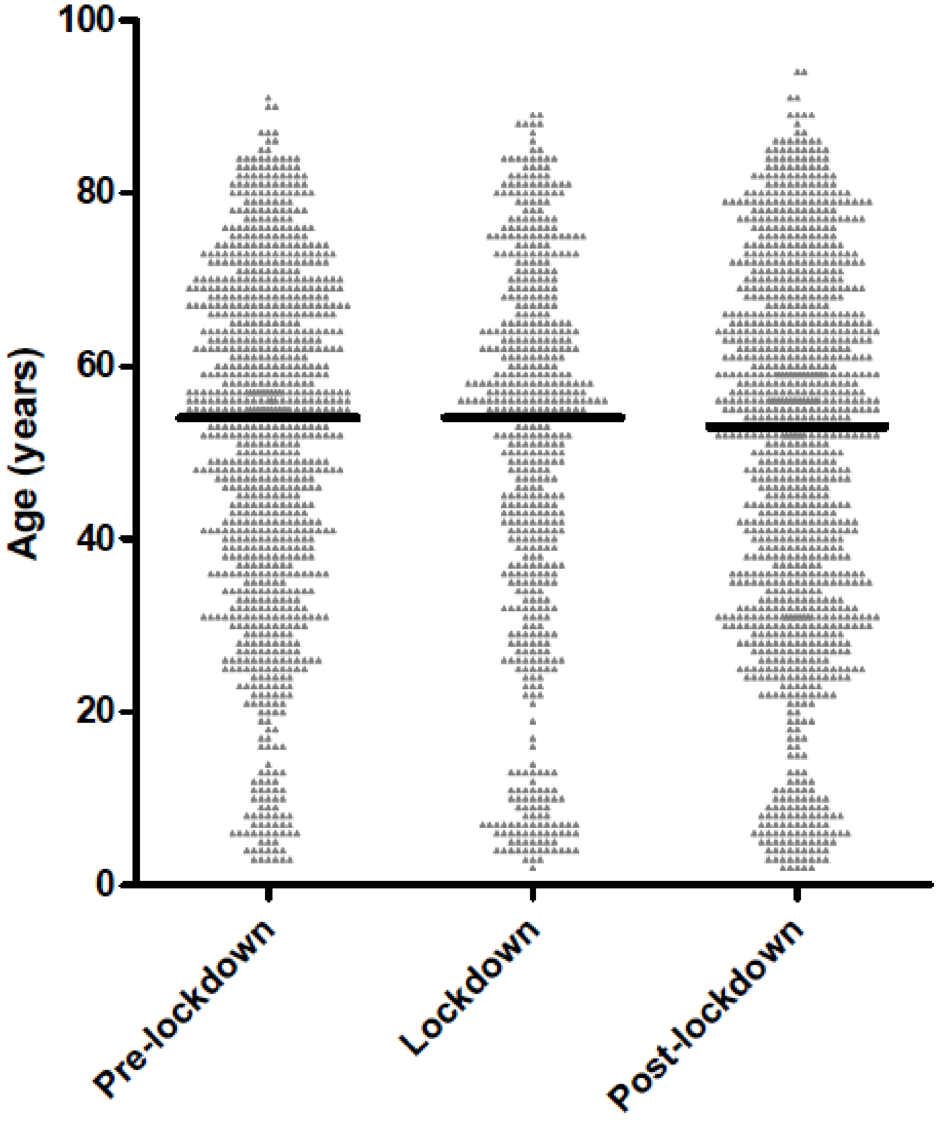

Figure S1. Dotplot of patients' age in urgent care pre-lockdown, during lockdown as well as post-lockdown.

Each dot represents one patient (n = 3014; median).
